# *Gripe-Needle*: A Sticky Suction Cup Gripper Equipped Needle for Targeted Therapeutics Delivery

**DOI:** 10.3389/frobt.2021.752290

**Published:** 2021-11-19

**Authors:** Kieran Joymungul, Zisos Mitros, Lyndon da Cruz, Christos Bergeles, S.M.Hadi Sadati

**Affiliations:** ^1^ School of Biomedical Engineering and Imaging Sciences Faculty of Life Sciences and Medicine, King’s College London, London, United Kingdom; ^2^ Wellcome/EPSRC Centre for Interventional and Surgical Sciences, University College London, London, United Kingdom; ^3^ NIHR Biomedical Research Centre at Moorfields Eye Hospital NHS Foundation Trust, UCL Institute of Ophthalmology, London, United Kingdom

**Keywords:** suction cup gripper, steerable catheters/needles, medical robots, bioinspiration, concentric tube robot

## Abstract

This paper presents a multi-purpose gripping and incision tool-set to reduce the number of required manipulators for targeted therapeutics delivery in Minimally Invasive Surgery. We have recently proposed the use of multi-arm Concentric Tube Robots (CTR) consisting of an incision, a camera, and a gripper manipulator for deep orbital interventions, with a focus on Optic Nerve Sheath Fenestration (ONSF). The proposed prototype in this research, called *Gripe-Needle*, is a needle equipped with a sticky suction cup gripper capable of performing both gripping of target tissue and incision tasks in the optic nerve area by exploiting the multi-tube arrangement of a CTR for actuation of the different tool-set units. As a result, there will be no need for an independent gripper arm for an incision task. The CTR innermost tube is equipped with a needle, providing the pathway for drug delivery, and the immediate outer tube is attached to the suction cup, providing the suction pathway. Based on experiments on various materials, we observed that adding a sticky surface with bio-inspired grooves to a normal suction cup gripper has many advantages such as, 1) enhanced adhesion through material stickiness and by air-tightening the contact surface, 2) maintained adhesion despite internal pressure variations, e.g. due to the needle motion, and 3) sliding resistance. Simple Finite Element and theoretical modeling frameworks are proposed, based on which a miniature tool-set is designed to achieve the required gripping forces during ONSF. The final designs were successfully tested for accessing the optic nerve of a realistic eye phantom in a skull eye orbit, robust gripping and incision on units of a plastic bubble wrap sample, and manipulating different tissue types of porcine eye samples.

## 1 Introduction

The challenge of actuating miniature forms of suction, especially in surgical settings has long been a researched topic. It is challenging to achieve viable solutions to adhere at such miniature scales whilst being able to incorporate interventional tools in a minimally invasive surgery setting. In this research, we propose a solution with a specific focus on the mechanical requirements of ONSF. We proposed a two-tube CTR equipped with a novel sticky suction cup gripper that houses a needle for therapeutics delivery in deep eye orbit. Inspirations from the octopus suckers have been sought to enhance the design robustness and adhesion capabilities.

**FIGURE 1:**
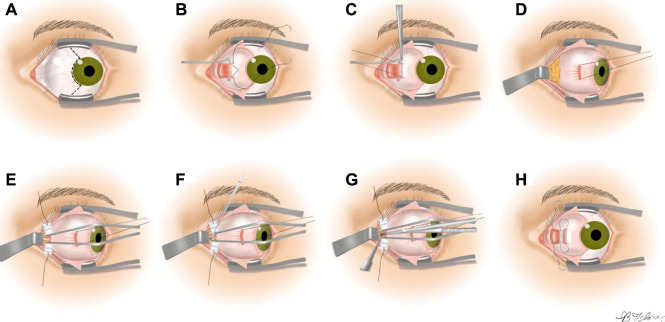
The steps of the medial transconjunctival approach (Mukherjee et al., 2013). **(A)** Performing peritomy, **(B)** medial rectus muscle isolation, **(C)** detaching the muscle from the globe, **(D)** retracting the globe laterally, **(E)** retracting the orbital fat from the optic nerve, **(F)** optic nerve sheath incision, **(G)** extension of the incision to 3–5 mm length, **(H)** reattaching the medial rectus and closing the peritomy.

ONSF is a surgically complex option to treat significant papilledema and the elevated hydrostatic pressure on the optic nerve when medication fails (Mukherjee et al., 2013). It involves surgically opening the optic nerve sheath to locally provide a sustained drop in intracranial pressure and relax “choking” the optical nerve. The preferred approach to ONSF is called“Medial Transconjunctiva”. However, To increase the operating field, the medial rectus muscle is separated from the eye globe via extensive manipulation of the eye which complicates the intervention and increases the chance of tissue damage. Therefore, ONSF is currently reserved for patients with significant papilledema and progressive or impending visual loss. Technique for performing ONSF are demonstrated in Figure 1 (Mukherjee et al., 2013).

**FIGURE 2:**
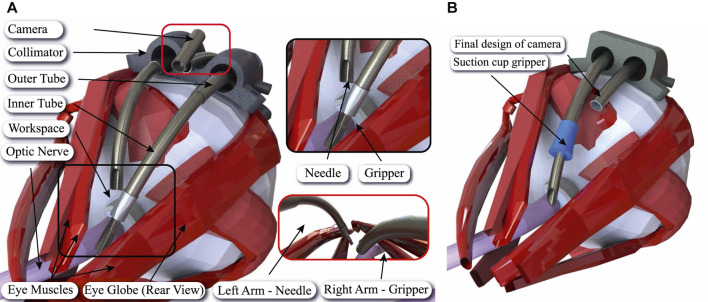
Sample application of the proposed sticky suction cup equipped needle design in ONSF. **(A)** Illustration of periocular access to the optic nerve (in pink) using the developed robot in Mitros et al. (2020) with three CTR arms (in dark gray) for incision, gripping, and camera motion. **(B)** Performing the same procedure with two CTR arms based on the proposed multi-purpose gripping (suction cup in blue) and incision tool-set design in this research. The new design is equipped with a smaller camera.

We have recently proposed accessing the eye orbit and the optic nerve by following the eye surface using a multiple Concentric Tube Robot (CTR) arms to collaboratively perform the intervention (Mitros et al., 2020) (see Figure 2A). A three-arm robot is proposed equipped with a camera, a needle, and a miniature gripper.

Each continuum arm is a CTR with three concentric tubes. The incision arm innermost tube is a sharp needle used for drug delivery. In this manuscript, we propose *Gripe-Needle*, a miniature suction cup gripper attached to the needle CTR arm to provide the gripping and incision functionalities at once. The tube cavities are separately used for drug delivery and maintaining the suction pressure needed for the gripper. As a result, smaller number of continuum arms are needed to perform the same ONSF task. This simplifies the robotic system design or provides the chance of equipping the extra CTR arm with alternative tools, such as surgical blades, to increase the setup capabilities (see Figure 2B). Our design is loosely inspired by the octopus’ suckers, grasping and boring technique for piercing sea snail shells.

To this end, a brief survey of the ONSF medical requirement and gripper designs in Minimally Invasive Surgery (MIS) with a specific focus on suction cup grippers and deformable cup-shape grippers are provided below.

### 1.1 Optic Nerve Sheath Fenestration Medical Requirements

The clinical literature on ONSF highlights the need of a vision module and at least two arms for tissue manipulation and completion of the surgical task; one arm holds the tissue with a micro-gripper while the other performs optic nerve cannulation. Workspace-wise, the surgical area of interest starts 2 mm posterior to the globe and extends posteriorly for a total length of 4–5 mm (Mukherjee et al., 2013). This workspace should be reached by following the curvature of the eye, therefore requiring the continuum arms to bend by at least that radius of curvature. To minimize disruption to surrounding tissue and to facilitate the insertion of the tools to the desired workspace, a bespoke collimator may be employed to introduce the continuum arms and hold the camera (see Figure 2A).

Our recent experimental studies based on a realistic silicon phantom and real porcine eyes showed that the eye globe can be safely deformed to make room for a device of up to 6 mm in thickness or outer diameter to reach behind the eye orbit. The incision force required for successful fenestration of the optic nerve was around 0.1 N (Mitros et al., 2020). The gripper should maintain a gripping force of the same magnitude in the normal and perpendicular direction of the incision to prevent the nerve sliding and retraction during the incision.

### 1.2 Medical Grippers for Minimally Invasive Surgery

Table 1 presents a summary of common gripping methods for MIS. The most common method of gripping a substrate for incisions or for manipulation is through the use of miniature pliers-like extremities (see Figure 2A). This is the most appealing method as it is simple in concept, relatively easy to use, tools are reusable and easy to disinfect. Moreover grip is easy to achieve and maintain to the operator’s discretion. Conversely, grip can often be hard to obtain for delicate procedures, the area in question where the substrate resides may be difficult to reach for the tool, and gripping can cause inadvertent tissue damage. As it is manually operated, gripping force relies on the surgeons expertise to maintain sufficient force. Even a soft grip over long duration may result in complications or prolonged healing process as more damage may be inflicted unintentionally.

**TABLE 1:** Comparison of the common gripping methods for MIS.

Method	Pros	Cons
Physical grip	Grip is easy to achieve, easy to maintain, and reusable	Grip area may be too close to the operating field. Pinching material will cause unintended local damages. It may be difficult to reach the target area due to limited space, dexterity, or manipulability. Grip relies on a constant applied force
Chemical adhesion	Causes little to no damage to substrate. Fairly reusable depending on the chemical material	Chemical used may not be able to achieve enough adhesion depending on the environment. Adhesion may wear off before expected. The effects of the chemicals used are likely to cause further complications. Complications due to permanent tissue adhesion
Sutures	Grip is definite and can be maintained for the duration of surgery. Dissolvable sutures could be used to minimise the complications	Tissue damage. Stitching is a time-consuming and complex and risky task requiring highly trained experts. Sutures may have to be cut post surgery to remove equipment. Complicats the surgical procedure. Not reusable
Suction Cup	Maintains adhesion due to pressure differences. No need for constant external pressure regulation. Reusable. The least invasive method amongst all	Likely to have little resistance to lateral motion on the substrate. Possible complex system design to achieve the pressure difference. Bulky design that prevents application for precise and narrow port surgeries

Suturing tissue and suturing equipment to substrates is another method of gripping substrates during surgical tasks. Sutures are readily available and achieves definitive grip for duration of operation or thereafter. Dissolvable sutures can be used to reduce harm and extension of healing. Sutures cause minimal damage to the substrates (Osterberg and Blomstedt, 1979). Although not often, it can be used to achieve a stable base for tools to be operated from, particularly to reduce the need for complex manipulation of tissues such as in (Mitros et al., 2020). There, suture is proposed to fix a collimator to the eye sclera in order to guide the miniature surgical robots and to reduce the procedure invasiveness (see Figure 2). However, it may be difficult and complex to suture equipment and tissue in the given intervention area. Sutures may also have to be removed, if dissolvable sutures are not a viable option, resulting in possible complications and damage.

Chemical adhesion is a less investigated method of achieving temporary grip for surgical proceedings. Medical adhesives are used to close both minor and major wounds along with suturing. Chemical adhesives have the potential to be used for temporary gripping tasks in MIS with good adhesive properties. This method causes little to no damage to the substrate, benefits from simple application which can reduce surgical duration, reduces the chance of some complications due to mechanical manipulation of the tissue, and lowers infection rates (Annabi et al., 2017). However, it may cause complications due to permanent tissue adhesion, may not achieve strong enough grips for anchoring surgical tools, or withstand interventional forces depending on the environment condition such as surface wetness and presence of body fluid.

Suction cup grippers are inspired by plethora of animals such as octopus, squid, frogs, and geckos. Suction cups have mainly been used for research, commercial and industrial applications to affix objects, manipulate delicate food products, anchor mobile robots to vertical surfaces, or for handling materials with nonporous surfaces in industrial settings (Mantriota and Messina, 2011; Sareh et al., 2017; Koivikko et al., 2021). However, deployment of the current suction cup designs in medical operations is challenging because of their large design and small sliding resistance if anchoring tools shifts base (Iwasaki et al., 2020).

### 1.3 Medical Suction Cup Grippers

The use of bioinspired suction cups for general medical applications with integrated actuation and sensing is proposed by (Sareh et al., 2017). More recently, (Iwasaki et al., 2020), have tested different suction cup designs for anchoring an untethered robotic capsules inside a patient’s stomach. They achieved the pressure difference through electromagnetic (EM) interactions between external control magnets and permanent magnetic elements inside the deformable cup body. The electromagnetic manipulation is used to navigate, reorient, regulate adhesion and peel off by controlling the torque experienced by the structure. However, the design is relatively large and adhesion is only maintained as long as electromagnetic torque is present that may result in patient discomfort.

### 1.4 Cup-Shaped Deformable Grippers

Deformable cup-shape designs for gripping general shape objects are proposed based on origami design. For example, Li et al. (2019) proposed that an origami “magic-ball” or“dragon egg”, comprised of many folds in a“water bomb” traversing pattern, can act like a gripping tool. Once sealed radially, such a design has the capability to exhibit a max volume contraction of 90% of its original to grip objects with general shape and size. However, the complex fabrication process poses challenges with miniaturizing such a design. Gripping flat and smooth surfaces would be challenging too.

Alternatively, Zhakypov et al. (2018) have proposed a universal gripper, for objects of any shape. A pyramid shape structure is designed with rigid vinyl plastic bits (forming the pyramid sides) embedded in a silicon structure (forming the flexible joints) that was actuated via thermoactive shape memory springs to morph the gripper opening to desired shapes. The hybrid rigid-soft gripper structure helps with maintaining the deformable structure directional rigidity for a robust grip. However, the proposed design was bulky with complex fabrication and actuation processes. As a result, it is challenging to design a miniature multi-material morphing origami structure for application in MIS.

### 1.5 Objectives and Novelties

This research is motivated by the previous work and inspired by the octopus suckers to design a minimally invasive suction cup that can house a robotic tool-set. We propose a miniature sticky suction cup gripper with a through access port where an interventional tool, e.g., a needle, is placed for simultaneous safe gripping the target tissue at the exact intervention vicinity (see Figure 2B). Gripping force is actuated through the cup sticky surface and manual suction vacuum. The gripped surface can be released by inducing positive pressure inside the cup or retracting the cup upon completion of the interventional task. The cup miniature design complies with the small access port of a CTR. The sticky surface air-tights the contact surface as well as provides lateral gripping force, a main disadvantage of current suction cup gripper designs.

To the best our knowledge, this is the first time that:

1. The multi-tube arrangement of a CTR is employed to actuate a multi-purpose tool-set,
2. A sticky suction cup gripper is proposed to maximize the gripping force in a miniature design and provides lateral gripping force,
3. A gripper with through access port is proposed for housing an interventional tool and securing the interventional site vicinity during the intervention.

In the remainder of this paper, we first discuss the biology that loosely inspired our multi-purpose tool-set design, a proof-of-concept gripper design, a miniature multi-purpose design incorporating an incision needle, and a simple theoretical framework to calculate the induced suction force, in Section 2. Section 3 presents the results from Finite Element Analysis (FEA), experimental studies and the parametric design for our suction cup designs. Results for 1) gripping and shear resistance force of the proof-of-concept and miniature cup designs based on different material surfaces, cup intact and grooved geometry, and in wet and dry conditions, 2) sample incision task on multiple units of a plastic bubble wrap sample, 3) accessibility test based on realistic eye and skull phantoms, and 4) porcine eye tissue manipulation and maximum gripping force statistics are reported. Discussions on the proposed design, further medical considerations, limitations, and our future plans are presented in Section 4.

## 2 Methods

Our design is loosely inspired by the octopus technique for grasping and boring sea snail shells, octopus sucker grooves to maximise the grip force, and its stickiness to boost sealing and surface contact. As a result, a proof-of-concept sticky suction cup gripper is designed and tested in pulling and shear tests on seven diffident material surfaces: natural rubber, latex rubber, rough surface wood, polished laminated wood, polished stainless steel, and 3D printed resin in dry and wet conditions. A simple theoretical framework is presented for calculating the gripping force and manual suction actuation pressure via a syringe to guide our miniature cup parametric design. FEA is introduced to investigate the gel and gripping surface deformations for different gripping stages (push-sealing, pulling, and near grip release), scenarios (wet and dry surfaces), and gel geometry (intact and grooved). Finally, a miniature multi-purpose design is discussed for a two-tube CTR comprising of a sticky suction cup gripper, a needle, and a small coaxial Y-junction for simultaneous control of the suction force, needle insertion, and drug delivery. The rest of this section provides more details on our research methods.

### 2.1 *Octopus* Sucker Biology, Snail Shell Grasping, and Boring Technique

Octopuses are incredibly adaptable and complex creatures with unique biological features. Most notably their suckers and ability to grip a variety of prey and surfaces offer incredible insight into feasible solutions for achieving adhesion at fine scales (Xie et al., 2020).

**FIGURE 3:**
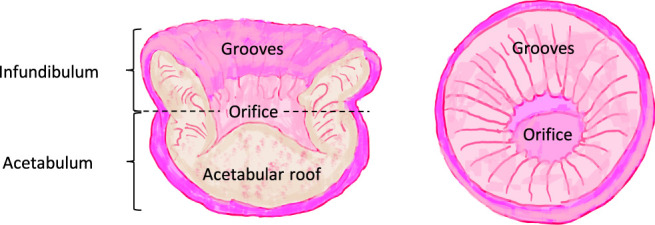
Structure of a typical octopus sucker, its cross sectional form and the two main features: the infundibulum and acetabulum (based on a drawing by Sareh et al. (2017)).

*Octopus* suckers are made up of two main areas, the infundibulum and acetabulum (Sareh et al., 2017) (see Figure 3). The function of the infundibulum is to provide a malleable, adaptable point of contact for the sucker such that it is able to induce an air-tight seal. The function of the acetabulum is to contract radially to reduce the pressure within the orifice and to create a large gradient between the sucker internal and surrounding pressure. As a result a relatively strong adhesion is achieved (Grasso and Setlur, 2007; Tramacere et al., 2013). Replicating the radial contractions of the acetabulum is challenging in a robotic design especially with miniature scales required for MIS applications. On the other hand, the sucker geometry, surface grooves, and possibly the surface adhesion-boosting properties can provide essential clues for such a design.

Each groove has a triangular cross-section starting from the orifice to the infundibulum. Grooves allow expansion and increase in the contact area of the infundibulum to induce a firm grip. Additionally, they allow easier sucker removal by directing the generated pressure gradient when the pressure gradient is reduced (Kier and Smith, 2002). The suckers’ surface material may contribute in the gripping efficiency. For example, mussel foot protein (mfp) is the adhesive protein involved in formation of a bundle of filaments that are important for anchoring sea mollusk muscles to various surfaces. Meloni et al. (2020) has recently proposed a bioinspired gripper design based on micro suckers coated with mfp solution to aid the wet surface adhesion. Their results suggest a significant increase in the adhesion properties in wet and moist environment for a coated surface with micro suckers (fabricated via direct laser lithography) compared to a flat one.

Furthermore, Octopuses and squids are both capable of boring holes into sea snails shells (Klompmaker and Landman, 2021). The cephalopods grip the sea snail with its beak. Once locked in place, the squid or octopus proceed to use its radula to apply pressure downwards into the shell whilst rotating clockwise and counterclockwise (Scheltema et al., 2003). The radula acts similar to a file tool that erodes the snail shell until it cut all the way through. At this instance, a mucus (immiscible with water) is passed into the hole that suffocates and forces the snail to leave the shell to get eaten.

*Octopus* suckers’ grooves and sticky surface, and the coaxial arrangement of the octopus beaks and radula while boring a sea snail shell have guided our multi-porpoise tool-set design in this research. To this end, a proof-of-concept gripper is designed to investigate the gripping performance of a sticky suction cup gripper on different material surfaces in wet and dry conditions.

### 2.2 A Sticky Suction Cup Gripper: Proof-of-Concept Design

**FIGURE 4:**
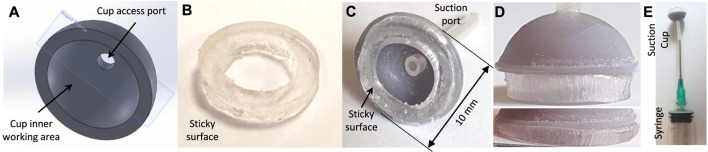
The proof-of-concept sticky suction cup design. **(A)** Cup CAD design, **(B)** sticky gel circular cut, **(C)** cup assembly, **(D)** gel undeformed and deformed side-view, **(E)** syringe assembly for inducing suction and grip release pressure.

A rigid structure was chosen to simplify the miniature fabrication issues, provide sufficient normal and lateral support, and avoid directional stability issues of a soft structure in our experiments. The cup structure (10 mm OD) was 3D printed with photopolymer (methacrylate monomers) resin. Figure 4 demonstrates the cup CAD design and assembly. A 10 ml plastic syringe was attached to the cup via a silicon tube to induce manual suction throughout our experiments. The parts were glued together with a resin glue to achieve the final assembly.

A piece of double-sided adhesive tape, washable transparent Acrylic gel, with 2.5 mm thickness was cut and attached to the cup base. The adhesive tape properties were 6 Kg/cm^2^ tensile adhesion, 3.9 Kg per 25 mm width 180° peel adhesion test (ASTM D-3330 standard), and 850 Kg/m^3^ density (www.psasolutions.uk.com). The tape was left to dry for a few days in room temperature and used after multiple cycles of attachment and detachment to minimise the effects that the experiment duration and repetition might have on the results. The tape’s strong tear resistance poses issues with a precise circular cut. Open ends of a stainless steel tube with desired diameters were sharpened and used to cut the tape circular shape. The picture of the cut gel was analysed using OnShape software to calculate the exact sticky surface and suction cavity area to be *a*_*c*_ = 66.6 and *a*_*p*_ = 31.7 mm^2^ respectively in undeformed (not squeezed against a material surface) state.

**FIGURE 5:**
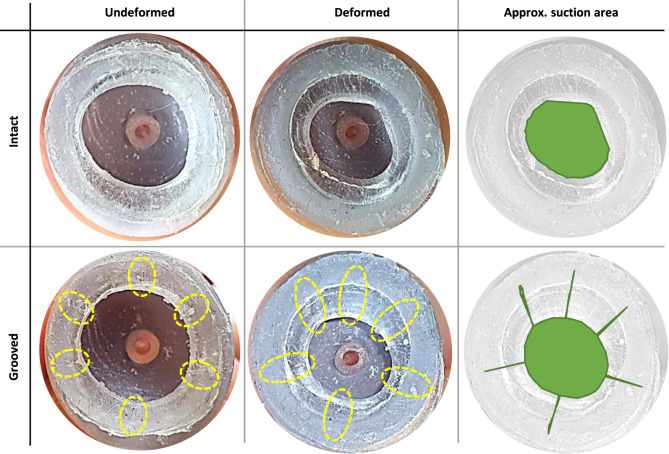
Effective suction area of compressed cup (against a clear glass) with intact and grooved (six grooves approximately 60° apart, marked with yellow dotted ellipses) sticky gels: approximate shape and area (shown in green). These figures are used to estimate the area correction factors C_c_ = 2.59 and C_p_ = 0.386 due to cup compression against a surface.

Six inclined cuts are introduced on the sticky surface with a sharp blade to mimic the grooves of an octopus sucker. The cuts are toward the central port leaving an intact thin but air-tight outer surface around the gel. Figure 5 shows the intact and grooved gel surfaces in relaxed and deformed states.

### 2.3 A Miniature Multi-Purpose Gripper and Needle Design

**FIGURE 6:**
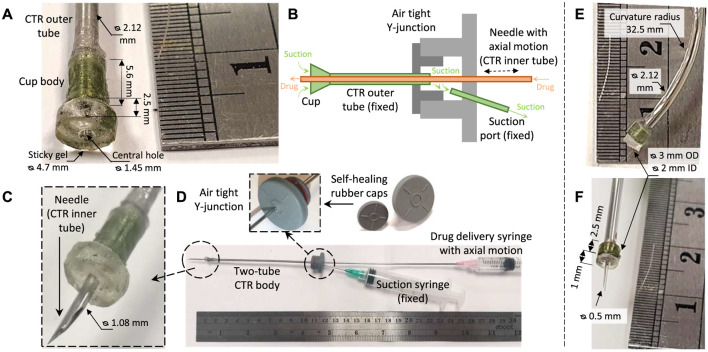
The miniature multi-purpose tool-set design consisting of a sticky suction cup, a needle, a small air-tight Y-junction, and the actuation syringes. **(A)** Cup assembly dimensions, **(B)** air-tight Y-junction working principle, **(C)** concentric needle incision equipped with the miniature suction cup, **(D)** system final assembly compatible with a two-tube CTR. A-D are used for grasping and incision task on a plastic bubble wrap sample. **(E)** A bent tube-fixed cup for eye orbit access validation. **(F)** A smaller cup and needle design for porcine eye tissue manipulation and incision experiments. Note the difference between the figure scales.

The final miniature design is consisting of a set two coaxial tubes (similar to a two-tube CTR) made of ultra-flexible nitinol tubes (0.88 and 1.5 mm ID, 1.08 and 2.12 mm OD for the inner and outer tubes respectively), a miniature sticky suction cup, and a small actuation/drug delivery Y-junction (see Figures 6A–D).

The cup small size makes precise 3D printing fabrication process challenging. As a result, the design was simplified to cutting the cone-shape syringe connection port of a commercially available needle to achieve the desired cross-section diameter for the miniature suction cup. The gel circular shape was cut using two stainless steel tubes with sharpened openings which were selected based on the desired gel circular shape radii. A larger design with 1.45 mm ID and 4.7 mm OD gel and 1.08 mm OD needle is used for incision test on a plastic bubble wrap sample (see Figures 6A–C). Two smaller versions of the cup (2 mm ID and 3 mm OD gel) were created: 1) with a curved outer tube for access feasibility study to the optic nerve (see Figure 6E) and 2) with a Microlance 3 needle (0.5 mm OD, as in the study by Mitros et al. (2020)) for porcine tissue incision tests (see Figure 6F).

Two Vial Butyl rubber caps with self healing and air-tightening properties and different 7 and 12 mm neck diameters were glued together (see Figure 6B) to form a compact design with an air-tight internal cavity. The outer nitinol tube was shrink fit inserted and glued to the smaller vial cap to form an air-tight stationary connection. The tube end remained inside the cavity to facilitate the cup suction actuation. A fine needle was inserted and glued to the side of the Y-junction to control the internal cavity suction pressure and consequently the suction cup through the outer tube opening. The inner nitinol tube equipped with a fine needle was inserted through the larger vial cap and passed through the CTR outer nitinol tube all the way through the suction cup gripper opening. The tube could slide while the vial rubber cap adequately air-tight the port. A syringe was connected to the inner tube for injection of the intended medication upon a successful incision. The tubes were not precurved similar to the configuration of CTRs, since the focus of our study was on the cup design and not the CTR positioning and control.

### 2.4 Finite Element Analysis and Theoretical Framework

FEA is used to investigate the gel complex deformation in different loading instances, surface conditions, and gel geometries. These analyses helped better understand the observed behaviour of the cup in the experiments (see the next section) and can guide the engineering design of the sticky gel in a future study. A simple theoretical framework is presented to model the cup suction pressure and overall gripping force.

**FIGURE 7:**
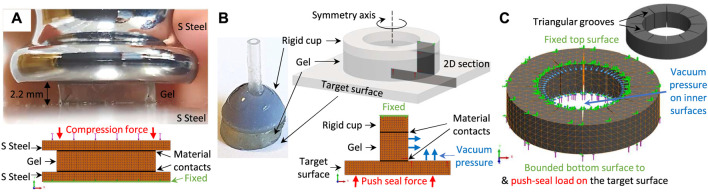
The FEA diagram for sticky gel elasticity modulus identification **(A)**, intact gel geometry simulation scenarios based on planar (2D) axisymmetric simplification **(B)**, and grooved gel geometry based on 3D simulations **(C)**, the target object is not displayed). The dimensions are as in Figure 4.

**Deformable Gel Finite Element Analysis:** A simple compression test based on a 10, ×, 10, ×, 2.2 mm gel sample was used to estimate the gel elasticity modulus *E*, while the material Poisson’s ratio and shear modulus are set to 0.48 and *E*/3 respectively. The FEA analysis was simplified to the case of planar stress case, due to the cube geometry planar symmetries. The gel was placed between two polished stainless steel surfaces and compressed under a heavy load while the deformation along the thickness is measured by post-processing the experiment video recordings. The material contact regions were modelled as bonded contacts in our FEA for the case of dry contact surfaces. To observe the gel deformation in low friction (slippery) surface conditions, in a separate experiment, the same gel sample was compressed once between wet polished stainless steel and smooth plastic surfaces. The material contact regions were modelled as impenetrable low frictional surfaces (with coefficient of friction 0.1) in our FEA for this case (see Figure 7A).

The identified values for *E* were used in FEA of the cups’ intact cylindrical shape gel (see Figures 4, 6). The cup gel symmetric loading and circular shape enabled us to simplify material domain to 2D axisymmetry cases. The gel top surface was fixed (via super glue to the rigid cup structure) while the bottom surface was in contact with the target object. This contact was either set as bonded (due to gel stickiness in the case of dry contact surfaces) or impenetrable but friction free (the case of wet contact surfaces). The gel inner area and the exposed part of the target object top surface were loaded with inward vacuum pressure. The target object bottom surface was loaded by a fixed normal force of 1 N too, only for the case of push-sealing the gel against the target surface (see Figure 7B). Both cases of rigid and soft contact target materials were investigated. Furthermore, three different deformable target materials were investigated with the same (same, *E*), half (softer, 50% *E*), and ten times (harder, 10 × *E*) the sticky gel stiffness. The simulations were carried out using simulation environment of SolidWorks software.

A 3-dimensional study is used to investigate the same scenarios for the gel grooved geometry where four, six, eight, and twelve cut-through triangular-shape grooves with 0.1 mm opening width were considered. The simulation is simplified by considering the gel only. A constant pressure equal to the suction pressure difference were applied to the gel inner area and the exposed section of the target surface. The top surface meshes, where the gel was glued to the rigid cup, was fixed. The target object bottom surface experienced a constant compression force of 0.5 N too, only for the case of push-sealing the gel against the target surface (see Figure 7C, target object is not displayed). The case of a deformable target surface with ten times higher stiffness than the gel were considered too.

Finally, a parameter study was carried out for the gel dimensions. Similar studies were carried out for the proof-of-concept cup as in Figure 4, based on a gel with half the proof-of-concept cup thickness and outer diameter, and for the miniature cup design as in Figures 6A,F.

**Gripping Force Theoretical Model:** A simple mathematical model is presented to predict the cup gripping force. The total normal (pulling) gripping force *F*_*n*_ is a summation of the surface stickiness *F*_*c*_ and the force induced by suction *F*_*p*_ as

$$F_{n}=F_{p}+F_{c}.$$ (1)

*F*_*c*_ is a function of the sticky surface area *a*_*c*_, the tensile adhesion stress *σ*, and the gel area expansion coefficient due to compression against a surface *C*_*c*_ as

$$F_{c}=\sigma C_{c}a_{c}.$$ (2)

*σ*_*c*_ (in Pa) is a function of the contact surface material and condition such as roughness and humidity, to be identified experimental. *F*_*p*_ can be calculated based on the suction cavity area *a*_*P*_, suction area shrinkage coefficient due to compression against a surface *C*_*p*_, pressure difference between the suction *P*_*c*_ and atmospheric *P*_0_ pressures, and suction force correction coefficient *C*_*f*_ as

$$F_{p}=C_{f}\left(P_{0}-P_{c}\right)\left(C_{p}a_{p}\right),$$ (3)

where *P*_0_ = 10^5^ Pa. *P*_*c*_ can be found based on the suction syringe change in volume Δ*V*, the gel expanded volume due to compression against a surface Δ*V*_*c*_, and the ideal gas relation in constant room temperature as

$$P_{c}=P_{0}V_{0}/\left(V_{0}+\Delta V-\Delta V_{c}\right),$$ (4)

where *V*_0_ = *V*_*p*0_ + *V*_*c*_ is the system initial volume which is a combination of the syringe initial state volume *V*_*p*0_ and the summation of the connection silicon tube and the cup internal cavity volume *V*_*c*_ ≈ 0.35 ml. For simplicity, all the experimental results are plotted against the syringe change in volume Δ*V*.

The cup shear (sliding) force resistance *F*_*s*_ can be found based on *F*_*n*_ and by defining a surface-specific static coefficient of friction *μ* as

$$F_{s}=F_{n}\mu .$$ (5)

The surface grip can be released upon inducing a positive pressure inside the cup, i.e. when Δ*V* < 0. In that case, a successful grip release is possible if *F*_*p*_ > *F*_*c*_.

As explained, multiple correction factors are considered to account for unmodelled phenomena. *C*_*c*_ and *C*_*p*_ accounts for the change in the stickiness and suction areas due to compression against a surface. *C*_*f*_ accounts for the reduced suction force due to peeling (non-uniform) detachment, leakages, non-ideal gas behaviour, and structural deformations induced by suction that results in higher internal vacuum pressure due to a smaller suction volume. The suction and gel stickiness area (measured) and correction factors (estimated experimentally) are assumed to be constant during the experiments. The presented model can be used for parametric design of a cup, e.g. calculating the contact surface outer and inner diameters based on the target surface material, the suction syringe volume range, and the desired gripping force. Results from our experimental and simulation studies are presented in the next section.

### 2.5 Experimental Studies

The proposed designs’ effectiveness were verified experimentally as described below.

**FIGURE 8:**
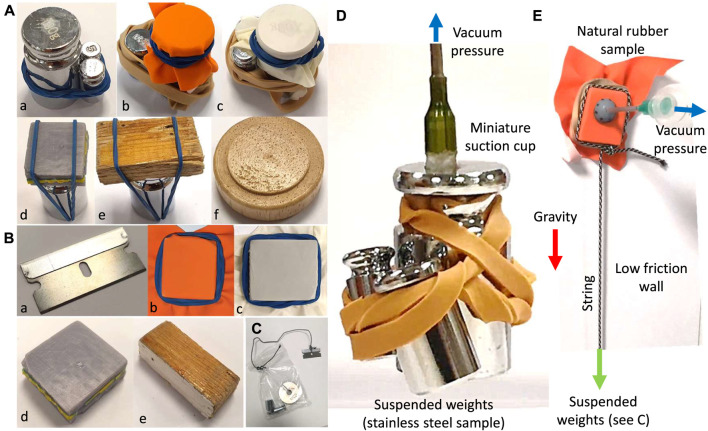
Sample surfaces used for pulling **(A)** and sliding **(B)** load bearing tests with the proof-of-concept cup design. Tested materials are polished stainless steel (a), natural rubber (b), latex rubber (c), 3D printed resin (d), polished laminated wood (e), and rough surface wood (f). The samples are either attached via a thread and a plastic bag **(C)** or wrapped (with a rubber band, **(D)** to different combination of calibration weights to estimate the cup load bearing in the shear and pulling experiments respectively. A sample pulling test with the miniature cup design **(D)** and shear test with the proof-of-concept cup designs **(E).**

**Pulling and Shear Force Experiments:** Pulling and shear load bearing tests are carried out using both the proof-of-concept (Figure 4E) and miniature (Figure 6F) cup designs on seven different material surfaces: rough surface wood, laminated wood, polished steel, natural rubber, latex rubber, paper, and 3D printed resin in both wet and dry conditions (see Figures 8A,B). Different surface materials are wrapped around a bundle of calibration weights to find the maximum load bearing of the design versus change in the suction syringe volume Δ*V*. For the shear tests, the surfaces were attached or wrapped around a thin flat substrate which was threaded to different combination of weights in plastic bag (see Figure 8C). A sample pulling test is shown in Figure 8D. For every test, the cup is firmly pushed against the surface for a perfectly air-tight seal before inducing the internal suction (see Figure 5). The masses are lifted until hanging free and exerting a force equal to their weight in both pulling and sliding tests. In the shear tests the cup was firmly pushed against the sample while it is held against a low friction vertical surface (see Figure 8E). The cup is slowly released after the desired vacuum pressure is induced. A successful grip is when the grip is maintained for approximately 15 s in both pulling and shear tests.

Deformable surfaces such as rubber and paper are attached via a rubber band to avoid constraining the material deformation that may result in an earlier grip failure due to the surface flexibility, e.g., via peeling. As a result, the experiments were more realistic by accounting both the material behaviour and surface properties. Calibration weights were used instead of a force sensor setup to test the successful release of the weights under gravity upon completion of each test. The weight of the attachments are measured and included in the reported values. The syringe initial volume *V*_*p*0_ was 0 ml for all the tests to achieve higher suction force via the syringe piston range.

**Effect of The Number of Grooves:** The effect of the number of grooves on the pulling and shear forces were investigated. To this end, multiple cups with intact and 4 to 12 equally spaced grooves were tested on a natural rubber surface sample in wet and dry condition.

**Miniature Cup Pulling and Shear Tests:** miniature cups were designed (see Figure 6) based on the results from experiments with the proof-of-concept cup design. The pulling and shear forces of the cup in Figure 6F with an intact gel and under maximum suction was tested on polished stainless steel and natural rubber surfaces, based on the procedure explained above. As a result, we could verify the miniature design to provide the required forces for ONSF (0.1 N) and identify the effect of miniaturization on the cup performance.

**FIGURE 9:**
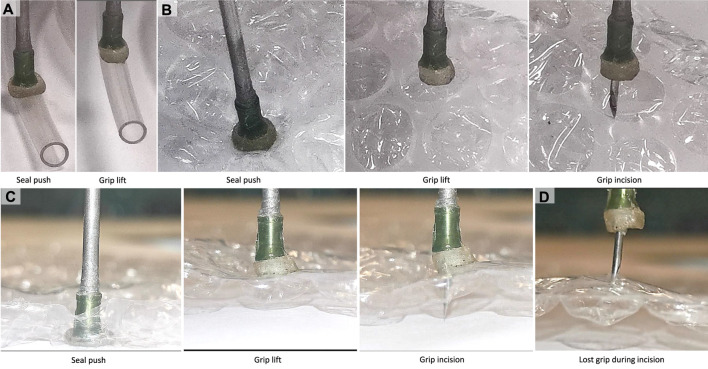
Experiments with the miniature multi-purpose tool-set design. Gripping and lifting a 4 mm OD silicon tube **(A)**, gripping, lifting, and piercing of a bubble unit **(B)** curve (dome) side, **(C)** flat side) of a plastic bubble wrap sample, and instance of occasional grip lose during a successful incision **(D)**.

**Grasping and Incision Tests on a Plastic Bubble Wrap:** Results from the load bearing experiments with the proof-of-concept design were used for parametric design of the miniature cup based on the force requirements of ONSF. The miniature suction cup in Figures 6A,C,D was successfully tested for gripping a silicon tube with 4 mm OD (see Figure 9A). To investigate the successful incision task, tests were carried out on multiple bubble units of a plastic bubble wrap sample from both the curved (doom) and flat sides of the units (see Figures 9B,C). A bubble wrap was selected because:

1. Grasping both a crumbled curved dome-shape surface an a completely flat and pre-stretched surface are challenging tasks for the state-of-the-art medical miniature grippers,
2. A successful incision can be easily verified visually due the transparent plastic material and based on the units’ internal pressure change post incision.

The miniature suction cup was firmly pushed against the surface for an air-tight seal before a maximum suction of Δ*V* = 10 ml was applied. The unit was slightly lifted and firmly shaken to test the grip robustness. Then, the unit was kept lifted while the needle was pushed through the sample and retracted. The grip is then released via increasing the cup internal pressure to compensate for the gel stickiness if needed (see Figures 9B,C). Results from the discussed simulation and experimental studies are presented in the next section.

**FIGURE 10:**
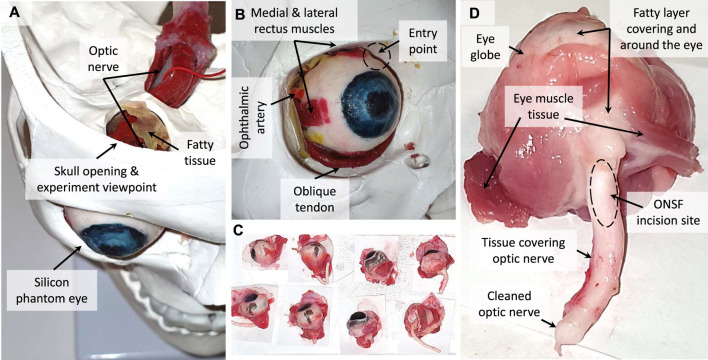
The silicon phantom **(A,B)** and real porcine **(C,D)** eyes used for accessibility and tissue manipulation performance of the miniature suction cups. The device entry point in the experiments and tissue types tested are showed in figures B and D respectively.

**Eye Orbit Accessibility Test:** The accessibility of the miniature cup in Figure 4F to the optic nerve in the eye orbit was successfully tested based on a high fidelity phantom eye orbit fabricated by Mitros et al. (2020) (see Figures 10A,B). The phantom eye were fabricated out of silicon and based on a segmented 3D MRI scan of an adult patient’s eye. Part of the phantom skull eye orbit was removed to view and record the procedure during the experiments. The miniature cup was connected to syringe to induce the necessary suction pressure via a bent tube (ID 1.5, OD 2.12, and curvature radius 32.5 mm) as in Figure 4F. The cup was successfully inserted through the space between the top of the phantom eye and the skull eye orbit to reach the optic nerve (white tube in Figure 10A). We manipulated the phantom optic nerve after push-sealing it against the nerve and inducing the vacuum pressure via the syringe.

**Porcine Eye Tissue Manipulation Tests:** The miniature cup and needle tool-tip in Figure 6F was tested for manipulation, pulling test via lifting the sample, and incision of 18 porcine eyes’ tissue, some of which are shown in Figure 10C. The porcine eye samples were collected not more than 2 days prior to the experiments and kept in cool enclosed plastic bags to maintain the natural tissue elasticity, malleability, and surface moisture. The tested tissues were 1) medial or lateral rectus muscles, 2) optic nerve, and 3) fatty tissue covering and surrounding the eye (see Figures 10D). The weight of the lifted samples were recorded as an indication for the cup maximum pulling force on each tissue type. The results for the theoretical and experimental studies are presented in the next section.

## 3 Results

In this section, the results of our experimental and simulation studies are presented. The load bearing capabilities of the proof-of-concept and miniature cup designs were evaluated based on FEA and experimental studies for pulling and shear tests on different material surfaces in dry and wet conditions. All the experiments are repeated three times and the averaged values are reported. The advantages of introducing grooves on the gel surface was briefly discussed. The results of these experimental studies were used to find the required outer diameter for a miniature sticky suction cup design capable of providing 0.1 N grasping and shear resistance forces. A set of miniature multi-purpose tool-sets were designed and the successful rate of performing simultaneous grasping and incision tasks on multiple units of a plastic bubble wrap sample, accessibility to the eye orbit, and success rate in manipulation of different real porcine eye tissue are reported.

### 3.1 Finite Element Analysis Results for Intact Gel Geometry

**FIGURE 11:**
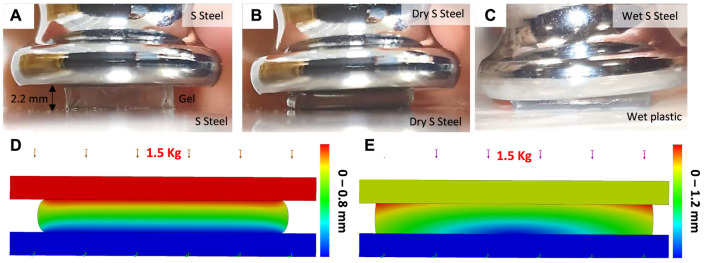
A 10.16 × 9.66 × 2.2 mm sticky gel cubic sample under compression test with ≈1.5 Kg load: undeformed case **(A)**, compression between dry stainless steel surfaces **(B)**, compression between wet stainless steel and plastic surfaces **(C),** FEA results for case **B** **(D)**, and case C **(E)**.

**FIGURE 12:**
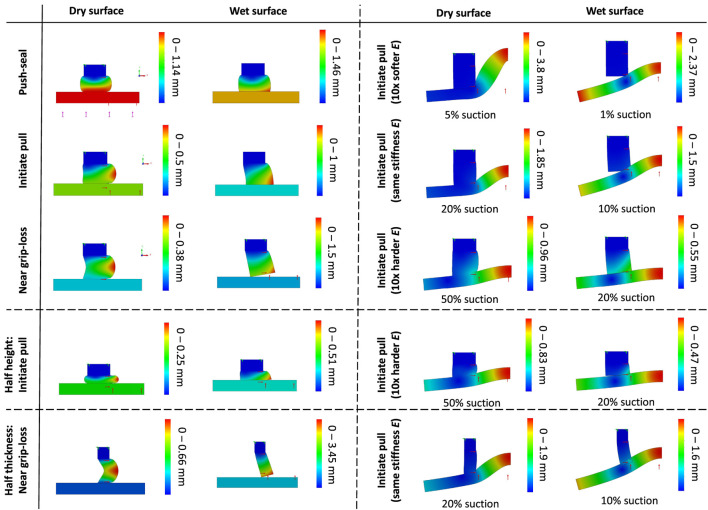
FEA results for an intact gel after 2D simplification due to geometrical and loading condition axisymmetry. The cup axis is on the right side. Simulations are carried out for different stages of a grip (push sealing to air-tight the cup, initiating pull when the target object weight is not opposing the suction grip force, and near grip loss when the suction grip force is fully compensated by the object weight), surface conditions (wet and dry), target material stiffness (rigid, 10× harder, 10× softer, and similar stiffness compared to the cup gel material), and parametric study (gel with half the height and thickness). Δ*p* = 1 bar suction pressure (100%) was induced unless specified otherwise.

Results based on FEA for material modulus of elasticity *E* identification, proof-of-concept, and miniature cups are reported in Figures 11, 12 respectively. Different instances of gripping, i.e., push-sealing, pulling, and grip release, on rigid and deformable surfaces in dry and wet conditions are investigated. A parametric study based on varying the height and thickness (i.e., outer diameter) of the proof-of-concept cup design are presented too. FEA results for the grooved gel geometry are discussed in the next subsections.

The dominant force among the suction and stickiness forces is expected to be the suction force, especially in the wet surface condition. Hence, a gel deformation resulting in larger suction area is desirable. The air tightening feature of the gel is maximised for a larger gel contact area. Furthermore, deformed gel geometries that result in an increase in the gel exerted force to the target surface boost the air-tightening feature. The FEA results are discussed based on the following criteria. In the deformed case under pulling force, an optimum gel geometry:

1. maximises the Cup Inner Suction Area
2. maximises the Gel Contact Area, and
3. passively pushes the gel against the target surface.

**Elasticity Modulus Identification:** A cubic sample of the gel experienced ≈0.8 mm deformation under 1.5 Kg compression load between dry polished stainless steel surfaces (see Figure 11A). As a result the modulus of elasticity was found to be ≈339 KPa (hence shear modulus to be 113 kPa, see Figure 11B). The same sample experienced larger 1.2 mm deformation and an ≈ 9% increase in the lateral deformation, but less stress concentration in the material for FEA in the case of wet surfaces. In the FEA, the maximum engineering lateral strain was ≈5.5 and 16% for the dry and wet surface cases respectively. These values were ≈7.5 and 26% in the FEA. This clearly shows that the material behaviour in the dry surface case is ≈2% and in the wet case ≈10% different from the ideal cases of bounded and friction-free contacts respectively. An estimate of the coefficient of friction between the surfaces can increase the FEA accuracy which is beyond the scope of this article. We disregard the material nonlinear, hysteresis, and creep behaviours in our FEA studies.

**Grip Stages:** Figure 12 presents the FEA results for the cup intact geometry in the different stages of grasping and releasing a rigid and deformable object in wet and dry surface conditions. The effect of changing the gel structural parameters are presented too. In the push-seal phase of a grip on dry rigid surface, the gel was pushed against the target object surface, expanding the area of gel contact (hence reduced the chance of grip loss), while reducing the suction area (and hence the grip force). Then, the cup was lifted slightly to initiate a lift when the weight of the target object was not contributing to the balance of the forces yet. As a result the gel contact area (and hence the air-tightening) was reduced while the gel was sucked further toward the cup center (symmetry axis) causing a reduction in the suction area and gripping force. The gel was still compressed due to the suction force on the target object. Finally, the case of near grip-loss was investigated when the suction force is balanced with the target object weight resulting in no further compression on the gel due to the suction force. As a result, the gel was further bent toward the cup center, further reducing the air-tightening and suction force. At this stage, the cup air-tightening is solely due to the gel stickiness (not considered in these analysis). The gel close angle with the target surface amplifies the air-tightening by pushing the gel against the surface due to the internal suction pressure. A sharper angle results in larger sealing force. Such an angle was present in all instances of the analysis.

**Wet vs. Dry Surface Conditions:** In the wet surface condition, the gel slides friction-free sliding on the target surface. As a result, the gel undergone larger deformation in all the instances of a grip resulting in a larger contact with stress concentration at the contact location with the surface. The gel larger deformation results in a larger contact area with the surface in the push-sealing and initiating a grip cases that increases the cup air-tightening. However, loss of gel stickiness to the surface resulted in the gel peeling off the surface in the near grip-loss case. Furthermore, a wide angle between the gel and target surfaces when initiating the pull may reduce the air-tightening by pulling the gel off the target surface. In all the instances, the gel was more deformed toward the cup center, reducing the suction area and hence the grip force. This can be negligible for a cup with large inner cavity but significant for a miniature one. A wet surface, resulted in lowering the grip force for miniature cups and easier grip-loss. However, A wet surface maximised the air-tightening feature by increasing the gel contact area.

**Rigid vs. Soft Target Surface:** A soft target surface deformed toward the cup center under suction. In the dry surface condition, this resulted in an increase in the gel contact area and hence air-tightening. However, a close contact angle did not form compared to a rigid target. Overall, the softer the subtrace was compared to the gel stiffness, the less the gel deformed. As a result, a softer subtrace could deform to a point that detaches from the gel and reduces the air-tightening in the wet surface condition. A harder subtrace prevented this detachment as the gel deformed according to the surface too. This emphasises the importance of choosing the gel stiffness to be softer than the subtrace for an optimum grip.

**Parametric Study:** To investigate the effect of the cup structural parameters, the gel height and thickness were halved while keeping the cup inner diameter (hence the suction area) fixed. The change of parameters did not significantly vary the observations with the original cup dimensions, except for the cases that are shown in Figure 12. A shorter gel (half the original height), resulted in smaller overall gel deformation, less creep toward the cup center, and even expansion away from the cup center when initiating a pull on a rigid target. As a result, it has less adverse effect on the suction cup area and pull force. On the other hand, a thinner gel (half the original thickness) amplified such a creep toward the cup center on rigid targets resulting in an even smaller suction area. Smaller contact area in this case has adverse effect on air-tightening too. However, the lower gel stiffness due to smaller thickness resulted in the better compliance and no detachment of the gel with a deformable subtrace and better air-tightening feature. As a result, unlike the original cup dimensions, an effective grasp could be achieved on a subtrace with similar elasticity modulus to the cup gel. Overall, a shorter cup can minimise the adverse effect of the gel deformation on the suction area, while the cup thickness can be tuned to achieve the desired overall gel stiffness based on the target surface elasticity.

### 3.2 Pulling and Shear Force Experiments

**FIGURE 13:**
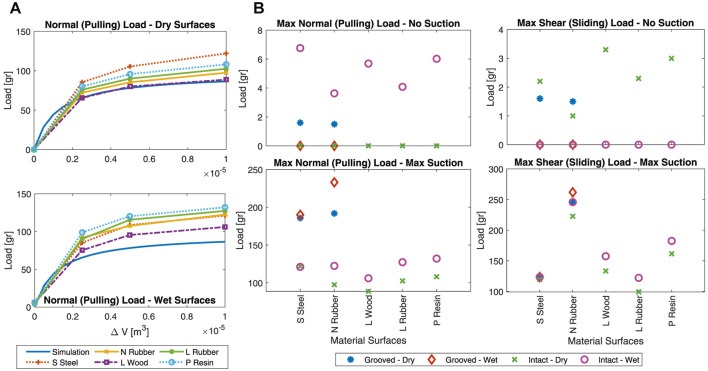
Load bearing test results on different surface materials in dry and wet conditions. **(A)** Normal (pulling) load bearing vs. change in the vacuum volume. Simulation results for suction-only grip load bearing is plotted to highlight the importance of gel stickiness. Middle) Maximum pulling, and **(B)** maximum shear load bearing due to intact (all surfaces) and grooved (S Steel and N Rubber surfaces only) gel stickiness. Results are reported for no vacuum and maximum vacuum (Δ*V* = 1 m^3^) pressure values. S Steel is polished stainless steel, N Rubber is natural rubber, L Wood is polished laminated wood, L Rubber is latex rubber, and P Resin is 3D printed resin surface material. Results for rough surface wood and paper are not plotted due to their negligible load bearing capability.

Figure 13 shows the results for the pulling and shear load bearing experiments on the different material surfaces in wet and dry conditions vs the change in the vacuum volume Δ*V*. Simulation results for *F*_*p*_ is plotted in comparison with the experimental results to show the effect of the gel surface stickiness *F*_*c*_ on the overall gripping force.

**Simulation Results and Parameter identification:** To find the correction factors *C*_*c*_ and *C*_*p*_, pictures were taken and analyzed for the cup when it was firmly pushed against and gripped (via 10 ml vacuum volume) a transparent glass surface (see intact deformed vs undeformed case in Figure 5). *C*_*c*_ is the ratio of the gel expanded to its undeformed area. *C*_*p*_ is the cavity shrunk to its undeformed area. These coefficients were found to be *C*_*p*_ = 0.386 and *C*_*c*_ = 2.59.

Δ*V*_*c*_ can be found based on the gel expanded surface inside the cup cavity (which was measured to be 84 mm^2^) and the deformed gel thickness (to be *t* = *t*_0_/*C*_*c*_ assuming the gel constant volume, where *t*_0_ = 2.5 mm is the initial thickness). The reduction in vacuum volume due to the gel deformation was found to be Δ*V*_*c*_ = 0.08 ml.

The cup showed no grip on the dry laminated wood without internal suction, i.e., no gel stickiness to the surface (*F*_*c*_ ≈ 0), and presented the least gripping force for the maximum suction value amongst all the tested surfaces. Furthermore, we assumed that smaller vacuum pressure values result in less change in the gel stickiness due to pressure, i.e., *F*_*c*_ remained almost constant (i.e., zero) for the laminated wood with Δ*V* ∈ [0, 25] ml suction volume. Hence, the pulling load results for this case with Δ*V* = 25 ml vacuum volume (*F*_*p*_ ≈ *F*_*n*_ = 0.64 N) was used to calculate the suction force correction coefficient to be *C*_*f*_ = *F*_*p*_/((*P*_*c*_ − *P*_0_)*a*_*p*_) = 0.78, where *P*_0_ = 10^5^ Pa, *a*_*p*_ = 31.7mm^2^, and *P*_*c*_ was derived based on Eq. 4. The coefficients *C*_*p*_, *C*_*c*_, *C*_*f*_, Δ*V*_*c*_, and Eq. s (one to four) are used plot the simulation results in Figure 13.

**Gel Stickiness Calculation:** Table 2 presents the gel stickiness *F*_*c*_ in KPa to different tested surfaces and for different vacuum volume and surface conditions. The values for *F*_*c*_ are calculated by subtracting the vacuum force *F*_*p*_ based on numerical simulations from the experimentally measured total normal (pulling) force *F*_*n*_ as *F*_*c*_ = *F*_*n*_ − *F*_*p*_. The absolute and relative difference between the values for different vacuum volume and surface conditions are reported too.

**TABLE 2:** Intact gel stickiness *σ* in KPa (maximum normal (pulling) force in N per initial gel surface area of 31.7 mm^2^) on different surfaces under no vacuum and maximum vacuum pressure. Changes in gel stickiness due to surface condition and vacuum pressure are reported too.

Surface	Vacuum	Polished	Natural	Polished	Latex	3D Printed
**Condition**	(Δ*V*)	**Stainless Steel**	**Rubber**	**Laminated Wood**	**Rubber**	**Resin**
Dry	Min (0 ml)	0	0	0	0	0
	Max (10 ml)	10.98	3.41	0.71	4.96	6.70
	Max-Min	10.98	3.41	0.71	4.96	6.70
	Max/Min %	NA	NA	NA	NA	NA
Wet	Min (0 ml)	2.09	1.12	1.76	1.26	1.86
	Max (10 ml)	10.72	11.12	6.05	12.64	14.12
	Max-Min	8.63	1.00	4.29	1.14	1.23
	Max/Min %	512%	990%	344%	1,001%	758%
Wet-Dry	Min-Min	2.09	1.12	1.76	1.26	1.86
	Max-Max	−0.26	7.71	5.34	7.67	7.42
Wet/Dry	Min/Min %	NA	NA	NA	NA	NA
	Max/Max %	98%	326%	851%	255%	211%

**Proof-of-Concept Design Performance:** The tested surfaces can be grouped as smooth (polished stainless steel and polished laminated wood), semi-smooth (3D printed resin, natural, and latex rubber), and rough surfaces (normal paper and rough wood). The cup showed no pulling load bearing in dry condition and very small load bearing in wet condition (4.8 gr for paper and 3.4–3.8 gr for rough wood) on the rough surfaces regardless of the gel stickiness and vacuum pressure. Hence, the shear grip tests were not carried out on this group of materials.

The pulling force increase by suction was higher for semi-smooth surfaces compared to the smooth ones while this increase was similar for the material within each category. The shear grip force increase by suction was similar for all the tested surfaces, except for the natural rubber which experienced a significantly larger increase. Observing the same grip force increase for the rigid 3D printed resin and deformable rubber layers, and different shear grip force increase between the deformable rubber layers showed that the material stiffness did not play a significant role on the effect of vacuum pressure on the cup load bearing. The highest gripping forces could be achieved on the semi-smooth surfaces. Overall, the cup total grip force *F*_*n*_ deviated more from the suction induced force *F*_*p*_ as the vacuum pressure increased. This showed that the gel stickiness increased with the vacuum pressure increase probably due to the increased effective contact area.

Wet surfaces had a smaller friction coefficient resulting in a smaller shear grip force for zero vacuum pressure cases. However, wetness revived the gel stickiness and improved the gel air-tightening feature on surfaces that could retain moisture (i.e., all the tested surfaces except polished stainless still) by sealing the gel layer gaps. As a result, we observed a larger pulling grip force with and without internal suction and a larger shear force with maximum suction. However, polished stainless still surface, which could not retain or absorb moisture, showed the largest pulling force increase when there was no internal suction (probably due to the gel increased stickiness to its smooth surface), but showed almost similar force value with the maximum internal suction. Shear grip forces were generally lower for dry surfaces and higher for wet surfaces, showing the importance of gel improved stickiness and air sealing on the cup shear load bearing.

### 3.3 Effects of a Grooved Sticky Surface

Figure 5 shows the difference in the deformation of a grooved sticky gel vs. an intact one. The gel had self healing properties making the grooves hardly visible and limited their effect to small dents on the gel surface. Grooves resulted in a smoother deformed surface, by reducing the stress concentration in the gel, and slightly increased the effective suction area. As a result, grooves could maintain the suction area when the cup internal area was reduced by the incision tool (needle) and helped effectively propagate the internal positive pressure to compensate for the gel stickiness when releasing the grip. However, grooves reduced the life cycle of the cups significantly as the cuts propagated through the external walls and damaged the gel air-sealing feature permanently.

**FIGURE 14:**
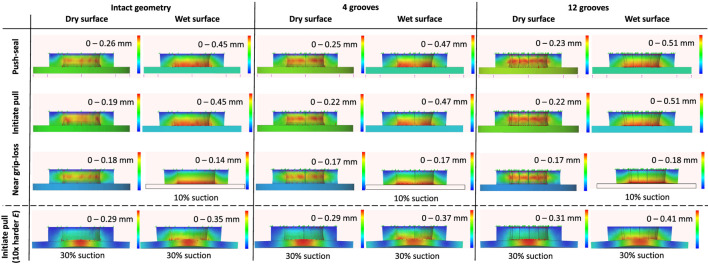
FEA results for intact and grooved (with 4 and 12 triangular cuts) gel. Simulations are carried out for different stages of a grip (push sealing to air-tight the cup, initiating pull when the target object weight is not opposing the suction grip force, and near grip loss when the suction grip force is fully compensated by the object weight), surface conditions (wet and dry), and target material stiffness with 10× harder stiffness compared to the cup gel material. Δ*p* = 0.5 bar suction pressure (50%) was induced unless specified otherwise.

**FEA Results:** Figure 14 presents the FEA results for intact gel geometry in comparison to grooved ones with four and twelve triangular cuts in different stages of a grip, on wet and dry surface conditions and for rigid and soft target surfaces. In the push-seal stage, the larger number of grooves resulted in smaller overall gel deformations on a dry surface but larger deformations on a wet one. This may indicate better air-tightening but larger reduction in suction area, and hence the grip force, of a grooved gel on wet surfaces. On the other hand, a the suction area was less affected by a grooved geometry on a dry surface, indicating higher suction force. These effects amplified for larger number of grooves.

A grooved geometry amplified the gel deformation when initiating the pull in both dry and wet surface conditions, resulting in better air-tightening but a reduction in grip force. Similar to the push-sealing stage, these effects amplified for larger number of grooves, but the difference is less significant on a dry surface. Furthermore, The gel deformation difference remained negligible between the two stages of a grip for the grooved geometries on a wet surface, while the gel deformation reduced after removing the push-sealing force on a dry surface This shows the superior sealing property of a grooved geometry on wet surfaces without a need for an initial push-sealing stage. The role of the grooves in channeling the suction pressure under the contact surface may be less significant for wet surface condition, given that the grooves closed down under the surface exerted by the target surface.

In the near grip-loss stage, grooves moderated the difference between the gel deformation on dry and wet surfaces. Smaller gel deformation on a dry surface and larger deformation on a wet one were observed. These show the grooved geometry may improve the air-tightening on wet but adversely affects it on a dry surface. On the other hand, grooves resulted in a larger decrease in the suction area on a wet surface that may lead to a decrease in the gripping force. These effects amplified slightly by increasing the number of grooves. The grooves did not prevent the peeling off effect that is observed in Figure 12 for this stage. However, they remained open in both dry and wet surface conditions increasing the suction area.

Finally, the difference between the deformation of an intact and grooved geometry for initiating a grip on a deformable surface, with modulus of elasticity ten times higher than the gel material one, was similar to the case of a rigid subtrace in the same stage. However, in general, a deformable subtrace resulted in larger deformations in the dry condition and smaller deformations in the wet condition.

**Intact vs. Grooved Gel Geometry in Experiments:** Figure 13 shows the effect of the grooves on the cup load bearing. Grooves improved the grip force on dry surfaces regardless of the suction pressure and on wet surfaces in presence of suction pressure. However, the grip force was reduced for wet surface conditions when there was no vacuum pressure. It showed that grooves were risky for wet surfaces if suction was lost, e.g. due to surface irregularities or rapid motion. Natural rubber showed larger increase in the grip force due to the grooves compared to the polished stainless steel surface.

Effect of Number of Grooves in Experiments

**FIGURE 15:**
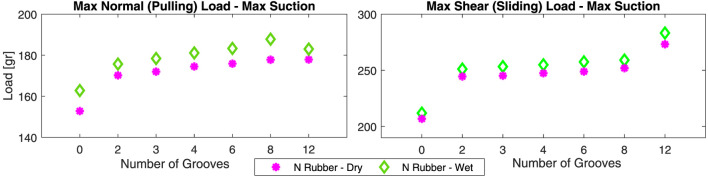
Normal and shear load bearing test results for cups with different number of grooves on natural rubber surface in dry and wet conditions.

Figure 15 presents the pulling and shear results on a natural rubber sample for cups with different number of grooves. As in Figure 13, forces were larger on wet surfaces, for grooved geometries compared to the intact one, and for the shear forces compared to the pulling forces. Presence of the grooves had the most significant effect on the forces while the change in the force values by increasing the number of grooves was less significant. The difference between the pulling forces in the wet and dry conditions were less significant for a grooved geometry compared to an intact one.

### 3.4 Miniature Gripper Parametric Design and Experiments

Experimental results based on the proof-of-concept cup design and the presented simple theoretical framework can be used for parametric design of a miniature cup given a griping task requirements.

**Cup Dimensions:** The miniature cup and gel ID should be larger than the needle OD of 1.08 mm. We chose the gel ID to be equal to the outer tube ID of 1.45 mm, to achieve a large sticky surface area while preventing the needle to cover the whole suction area during an incision. The results for the gel stickiness on different surfaces *σ* in Table 2 were used to calculate the miniature cup OD. We assumed the ONSF requirement of *F*_*n*_ = *F*_*s*_ = 0.1 N (normal and lateral griping force values) as in (Mitros et al., 2020) for our design. Assuming the maximum suction volume change of Δ*V* = 1 ml, the vacuum pressure from Eq. 4 is 3.41 KPa. Thus, the suction force during an incision task from Eq. 3 is 0.021 N. Based on Eq. 1, the gel stickiness should account for *F*_*c*_ = 0.1–0.021 = 0.079 N under maximum vacuum to maintain the required grip. We used Eq. 2 to find the gel OD. The ONSF environment is more similar to our wet surface condition experiments. We assumed values for *σ* based on measurements for laminated wood (6.05 KPa) in our calculations, since it provided the least gripping force under the maximum suction for a wet surface in our experiments. The gel OD is found to be ≈3 mm from Eq. 2. The required grip shear force of 0.1 N is achievable using these dimensions, since compared to the normal forces, larger shear forces were observed during our experiments for maximum suction value and wet surface condition. The final miniature designs used a 3 mm (for accessibility and porcine eye tissue manipulation tests) and a larger 4.5 mm (for plastic bubble wrap incision tests) OD cup. A larger cup is designed to account for errors in the modeling assumptions, identification procedure, and differences between the tested material and the target tissue properties.

**FIGURE 16:**
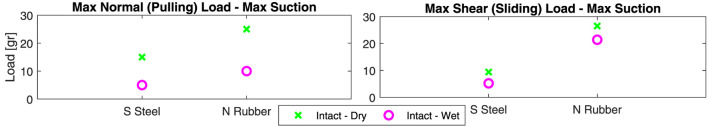
Normal and shear load bearing test results for the miniature cup in Figure 8D on polished stainless steel and natural rubber surfaces in dry and wet conditions.

**Miniature Cup in Pulling and Shear Experiments:** The results for the pulling and shear experiments (see Figure 8D) with the miniature cup with 3 mm OD (see Figure 6F) are presented in Figure 16. The cup could provide the required 0.1 N force for ONSF in both the normal and shear directions on the natural rubber surface regardless of the condition and on the polished stainless steel in the dry condition. The forces are smaller on the wet surfaces despite our observation based on the proof-of-concept cup design (see Figure 13).

This can be explained based on our FEA results (see Figure 12). On a wet surface, the gel deformation was larger. This may improve the cup air-tightening by increasing the contact area, but reduces the suction area and hence the gripping force. Our experimental results showed that the increase in the air-tightening feature is more dominant for a large cup while the reduction in the gripping force is more dominant for a miniature one. As seen in the FEA results, this can be addressed by employing a shorter gel. As a result, a 1 mm in thickness gel were used for the miniature cup that were used for manipulation tests on the real porcine eye tissue (see Figures 6E,F) which was more similar to our experiments in the wet surface condition.

**Sample Grasping and Incision Tasks Results:** Tests were carried out using the miniature cup design for simultaneous grasping and incision of multiple bubble units on a plastic bubble wrap sample. The miniature cup was successful in gripping both the curve (doom shape) and flat side of a bubble unit robustly even when the system was rapidly shaken. The grip could not be maintained during a needle incision task for an intact gel, i.e., without grooves, showing the importance of the grooves in a compact design.

We performed 50 gripping trials with the cup, including tests to debug the design issues and fine tune our gripping and incision techniques, before the gel was permanently damaged. A successful grip is defined as lifting a bubble unit and maintaining the grip during multiple rapid shakes of the device. A successful incision is identified upon entry of the needle into the bubble unit, regardless of the state of the grip. We recorded the successful rate of maintaining a grip during an incision task and after fully retracting the needle. Table 3 presents the results for the different stages based on the tests on the flat side of the bubble units, showing 81% incision and 75% maintained grip success rates, as the two most important tasks during such an intervention. The results for similar experiments on the curve side of the bubble units were about 30–40% less successful for gripping, incision, and maintained grip cases. However, we couldn’t maintain the grip post needle full retraction in any of these experiment instances. These results showed the importance of considering the grasping target shape in designing the cup sticky surface.

**TABLE 3:** Success rate for the miniature multi-purpose design of Figure 4A in gripping, incision, and maintaining grip during incision for experiments on a plastic bubble wrap sample. Percentage values are reported w.r.t. the total number of eight trials (see Figure 6).

Successful Grip Lift	Successful Incision (Regardless of grip lose)	Grip Maintained During Incision	Grip Maintained After Needle Retraction
8 (100%)	7 (81%)	6 (75%)	4 (50%)

**FIGURE 17:**
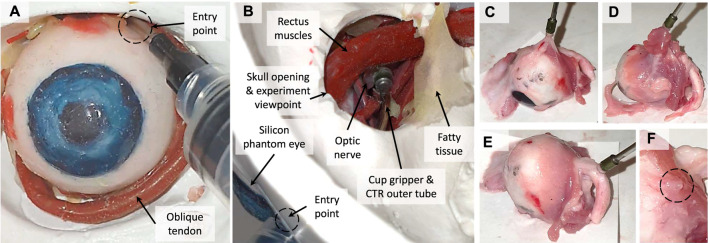
The accessibility to the optic nerve based on experiments with the miniature cup in Figures 4E, A realistic skull and silicon eye phantom: front **(A)** and top **(B)** views. Porcine eye tissue manipulation and maximum pulling force experiments with the miniature cup in Figure 4F: fatty layer and tissue **(C)**, muscle tissue **(D)**, cleaned optic nerve **(E)**, and temporary residual tissue deformation after a grip **(F)**.

**Optic Nerve Access:** Figures 17A,B show that the miniature cup in Figure 4E can successfully bypass the eye globe and reach the optic nerve in the skull eye orbit. A collimator as in Figure 2 temporarily sutured to the eye globe will be used to fix the eye motion during the real procedure, which is not depicted here.

**Porcine Eye Tissue and Optic Nerve Manipulation:** Figures 17C,F presents instances of experiments on manipulation, incision, and pulling experiments on different tissues (fatty layer and tissue, muscle tissue, and cleaned optic nerve) of multiple porcine eye samples (see Figure 10). We repeated each manipulation, incision, and pulling task at least five times before reporting it unsuccessful. In other words, a task was considered successful when it was achieved at least once in up to five trials. This is to minimise the effect of the variations of the tissue geometry, stiffness, surface moisture, the cup approach angle and initial push force between the eye samples and experimental trials on the success rate results.

**FIGURE 18:**
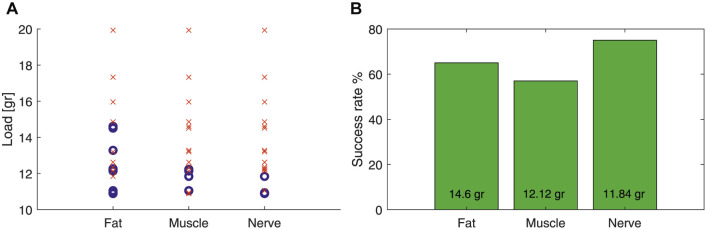
**(A)** Successful (blue circle) and unsuccessful (red cross) instances of lifting a porcine eye sample with the miniature cup in Figure 4F. **(B)** Success rate for lifting a sample with a mass up to the maximum reported value in the left plot.

The cup was 100% successful in manipulating the muscle and fatty tissue while failed only once to manipulate the optic nerve (95% success rate) in our trials with 18 porcine eye samples. Figure 18A shows that the cup could lift up to 14.6 gr (9 out of 14 instances, 65% success rate) when grasping a fatty tissue, 12.14 gr (4 out of 9 instances, 57% success rate) for muscle tissue grip, and 11.84 (3 out of 4 instances, 75% success rate) for optic nerve tissue grip. However, there were instances of grip loss for smaller force values. For instance, there were five instances (35% failure rate) of grip loss with loads as low as 11.84 gr when grasping a fatty tissue, 3 instances (43% failure rate) with loads as low as 10.89 gr for muscle tissue grip, and 1 instance (25% failure rate) with 11.05 gr load for optic nerve grip. Figure 18B presents the success rate for the reported maximum pulling force of each tissue type in our experiments.

The muscle tissue had the highest uncertainty in defining a maximum pulling force, probably due to its irregular surface shape. A clean optic nerve was the most difficult to grasp due to the smooth round surface geometry and stiffer tissue property. Tissue malleability could improve the pulling force significantly, as a result the fatty tissue presented the highest pulling force. Figure 17F shows the residual deformation in the grasped tissue after release. The deformation was temporary and gradually (over a few seconds) returned to its original stretched state.

## 4 Discussion

The presented results in the previous section shows the potential advantages and successful deployment of a miniature needle that is equipped with a sticky suction cup gripper for precise therapeutics delivery in MIS. We observed that a sticky suction cup may not provide good adhesion on rough surfaces, but semi-rough surfaces can be gripped effectively with significant increase in the gripping force via increasing the cup suction. Material rigidity did not contribute much in the cup performance, while surface wetness could enhance the vacuum grip in both pulling and shear loading tests.

A bioinspired grooved surface were found to be advantageous in vacuum grip cases for maintaining the grip during the incision task. Grooves, regardless of their number, could slightly increase the cup adhesion due to the increased suction area with larger effect on grasping semi-rough surface of a deformable natural rubber sample compared to the polished smooth surface of a rigid stainless steel one. However, the grooves significantly reduced the life cycle of the cup by deteriorating the air-sealing property of the gel.

FEA results for the proof-of-concept cup design showed the advantages of a wet surface and grooved geometries in increasing the gel air-tightening, but at the cost of reducing the suction area due to the larger gel deformations. These effects may increase the performance of a larger design while affect a miniature-size one. Reducing the gel height showed to reduce these adverse effects. The proposed simple model and the tests based on the proof-of-concept cup design were employed for parametric design of a miniature cup based on the force requirements of ONSF.

The miniature cup could successfully access the optic nerve of a realistic silicon eye phantom in a skull eye orbit. The grip, incision, and maintained grip during incision experiments showed the design to be highly successful (75–100%) in tasks involving a pre-stretched flat surface, but 30–40% less successful in tasks on a curved crumbled one. The effectiveness of the miniature cup design in the manipulation of optic nerve, fatty, and muscle tissues of multiple porcine eye samples were demonstrated while showing the challenges in maintaining a grip during incision on a real tissue. The observed minimum grip force values on different real tissue were larger than the ONSF requirement of 0.1 N (≈10 gr). However, the grip could not be maintained effectively during the needle incision. The design limitations and suggestions for future research are discussed in the following subsections.

### 4.1 Limitations and Future Directions

A number of limitations and drawbacks were identified during our experiments with the proposed proof-of-concept and miniature suction cup designs. Some of these limitations and suggestions to address them are listed below.

1. A sticky cup relies on positive internal pressure to release a grip. Light highly deformable surfaces tend to resist detachment by remaining partially attached to the cup.
2. Rough surfaces, curved geometries, and surfaces that are deforming during intervention (incision) proved to be challenging to grip.
3. Despite the many advantages compared to the alternative medical micro gripper designs, a sticky suction cup requires an initial firm push against a surface to fully seal the internal cavity. As a result, it is challenging to grip suspended and floating tissue.
4. The cup’s internal tool motions and interactions with the target surface affect the cup performance.
5. The compact Y-junction design was successful in providing an air-tight coaxial connection with translational motion, but couldn’t provide a robust support for the cup motion especially to support the initial push against the surface.
6. We observed some discrepancies between the performance of different cups on polished stainless steel surface, probably due to the gel surface dissimilarities.
7. Despite the successful experiments on accessibility and tissue manipulability, our simultaneous incision and gripping trials were mostly unsuccessful, given the small size, relatively stiff material, and cylindrical shape of the cup gel.

**Suggestions to Improve The Cup Design:** The cup design can be improved to address some of the aforementioned limitations. The gel material geometry and material properties such as elasticity, stickiness, thickness, and surface profile (e.g., inclined or curved) can be fine tuned to better conform with rough surface material and curved geometries. Optimizing the groove geometry can help better distribute the suction and positive internal pressures for firmer grasp and more effective detachment of highly flexible surfaces. A cup with long thin sticky edges can be tested to help with the initial sealing stage and reduces the need for an initial firm push against the grip surface. A commercially available multi-port introducer sheath, such as Gore DrySeal Flex, with sealing functionality for a translational guidewire tools and micro valves to prevent leakage of the drug fluid in the suction cup area can replace the proposed compact Y-junction design. The use of more precise measurement equipment such as a micro force sensor and precision linear stage can help better understand the variability of the different cup performances. FEA-based optimization of a miniature cup can enable us to achieve the simultaneous incision and gripping on the real tissue similar to the plastic bubble wrap samples. Ideas regarding multi-layer, soft, origami structure cup designs can be investigated to address the identified limitations.

**Medically Safe Material and Sterilization:** In designing a medically safe device, material safety and sterilization requirements should be considered. A disposable design with medically graded silicone, a commonly used material in similar research (William Luria, 2002), is the most suitable choice for fabricating both the cup structure and deformable surface. Silicone rigidity can be easily tuned based on the basic components, mixing ratio, and curing procedure to fabricate the different parts of our proposed design. Medically safe glues, adhesives, or topical coagulants are widely used for wound closure, fluid leakage sealing, vascular embolization, and surface adhesion in surgery Dimitrakakis et al. (2011). Such material can be diluted to achieve the temporary adhesion requirements of a sticky suction cup gripper for medical applications and added (brushed or embed in a medically safe silicon gel structure) on the suction cup deformable surface.

### 4.2 Conclusion

In this research, for the first time, we utilized the multi-tube structure of a CTR to design a multi-purpose gripping and intervention tool-set. The proposed design is a miniature needle equipped with a sticky suction cup gripper, that we called *Gripe-Needle*, for precise therapeutics delivery in MIS. We showed that a sticky gel surface with bioinspired grooves can improve the sealing and shear force resistance of a miniature suction cup, even on wet surfaces, while highlighting the miniaturization challenges. Finite Element Analysis, a simple theoretical framework, and a set of experimental data are provided for similar suction cup designs based on the grip force requirements of a task. Suggestions for an improved design based on general medical requirements and *ex-vivo* experiments on porcine optic-nerve are proposed to further improve the design toward real-life deployment in an ONSF procedure.

## Data Availability

The raw data supporting the conclusions of this article will be made available by the authors, without undue reservation.

## References

[B1] Annabi N. Zhang Y.-N. Assmann A. Sani E. S. Cheng G. Lassaletta A. D. (2017). Engineering a Highly Elastic Human Protein-Based Sealant for Surgical Applications. Sci. Transl. Med. 9, eaai7466. 10.1126/scitranslmed.aai7466 28978753 PMC11186511

[B2] Dimitrakakis G. Podila S. R. R. O'Keefe P. A. Kulatilake N. E. P. (2011). Biological Glue: a Word of Careful Assessment!. Interactive CardioVascular Thorac. Surg. 13, 244–245. 10.1510/icvts.2011.273094A 21775494

[B3] Grasso F. W. Setlur P. (2007). Inspiration, Simulation and Design for Smart Robot Manipulators from the Sucker Actuation Mechanism of Cephalopods. Bioinspir. Biomim. 2, S170–S181. 10.1088/1748-3182/2/4/S06 18037726

[B4] Iwasaki H. Lefevre F. Damian D. Iwase E. Miyashita S. (2020). Autonomous and Reversible Adhesion Using Elastomeric Suction Cups for *In-Vivo* Medical Treatments. IEEE Robot. Autom. Lett. 5, 2015–2022. 10.1109/LRA.2020.2970633

[B5] Kier W. M. Smith A. M. (2002). The Structure and Adhesive Mechanism of Octopus Suckers. Integr. Comp. Biol. 42, 1146–1153. 10.1093/icb/42.6.1146 21680399

[B6] Klompmaker A. A. Landman N. H. (2021). Octopodoidea as Predators Near the End of the Mesozoic Marine Revolution. Biol. J. Linn. Soc. 132, 894–899. 10.1093/biolinnean/blab001

[B7] Koivikko A. Drotlef D.-M. Sitti M. Sariola V. (2021). Magnetically Switchable Soft Suction Grippers. Extreme Mech. Lett. 44, 101263. 10.1016/j.eml.2021.101263 33834089 PMC7610552

[B8] Li S. Stampfli J. J. Xu H. J. Malkin E. Diaz E. V. Rus D. (2019). “A Vacuum-Driven Origami “Magic-ball” Soft Gripper,” in 2019 International Conference on Robotics and Automation (ICRA), 7401–7408. 10.1109/ICRA.2019.8794068

[B9] Mantriota G. Messina A. (2011). Theoretical and Experimental Study of the Performance of Flat Suction Cups in the Presence of Tangential Loads. Mechanism Machine Theor. 46, 607–617. 10.1016/j.mechmachtheory.2011.01.003

[B10] Meloni G. Tricinci O. Degl’Innocenti A. Mazzolai B. (2020). A Protein-Coated Micro-sucker Patch Inspired by octopus for Adhesion in Wet Conditions. Sci. Rep. 10, 15480. 10.1038/s41598-020-72493-7 32968184 PMC7511962

[B11] Mitros Z. Sadati S. M. H. Seneci C. A. Bloch E. Leibrandt K. Khadem M. (2020). Optic Nerve Sheath Fenestration with a Multi-Arm Continuum Robot. IEEE Robotics Automation Lett. 5, 4874–4881. 10.1109/lra.2020.3005129 PMC7610935 34109274

[B12] Mukherjee N. El-Dairi M. A. Tariq Bhatti M. (2013). Optic Nerve Sheath Fenestration–Indications and Techniques. US Ophthalmic Rev. 6.

[B13] Osterberg B. Blomstedt B. (1979). Effect of Suture Materials on Bacterial Survival in Infected Wounds. An Experimental Study. Acta Chir Scand. 145, 431–434. 539325

[B14] Sareh S. Althoefer K. Li M. Noh Y. Tramacere F. Sareh P. (2017). Anchoring like octopus: Biologically Inspired Soft Artificial Sucker. J. R. Soc. Interf. 14, 20170395. 10.1098/rsif.2017.0395 PMC5665824 29070591

[B15] Scheltema A. l. H. Kerth K. Kuzirian A. M. (2003). Original Molluscan Radula: Comparisons Among Aplacophora, Polyplacophora, Gastropoda, and the Cambrian fossilWiwaxia Corrugata. J. Morphol. 257, 219–245. 10.1002/jmor.10121 12833382

[B16] Tramacere F. Beccai L. Kuba M. Gozzi A. Bifone A. Mazzolai B. (2013). The Morphology and Adhesion Mechanism of *Octopus vulgaris* Suckers. PLOS ONE 8, e65074. 10.1371/journal.pone.0065074 23750233 PMC3672162

[B17] William Luria L. (2002). The Role of Medical Grade Silicones in Surgery and its Topical Applications. Oper. Tech. Plast. Reconstr. Surg. 9, 67–74. 10.1016/S1071-0949(03)90012-6

[B19] Xie Z. Domel A. G. An N. Green C. Gong Z. Wang T. (2020). Octopus Arm-Inspired Tapered Soft Actuators with Suckers for Improved Grasping. Soft Robotics 7, 639–648. 10.1089/soro.2019.0082 32096693

[B20] Zhakypov Z. Heremans F. Billard A. Paik J. (2018). An Origami-Inspired Reconfigurable Suction Gripper for Picking Objects with Variable Shape and Size. IEEE Robot. Autom. Lett. 3, 2894–2901. 10.1109/lra.2018.2847403

